# Smartphone addiction in children: patterns of use and musculoskeletal discomfort during the COVID-19 pandemic in Iran

**DOI:** 10.1186/s12887-022-03748-7

**Published:** 2022-11-26

**Authors:** Hamid Reza Mokhtarinia, Maryam Heydari Torkamani, Ozra Farmani, Akbar Biglarian, Charles Philip Gabel

**Affiliations:** 1 Department of Ergonomics, University of Social Welfare and Rehabilitation Sciences, Kodakyar Ave. Daneshjo Blvd, Evin, PC: 1985713834 Tehran Iran; 2 Iran Welfare Organization, PhD of Social Work, Tehran, Iran; 3 Department of Biostatistics and Epidemiology, Social Determinants of Health Research Center, University of Social Welfare and Rehabilitation Sciences, Tehran, Iran; 4 Access Physiotherapy, Coolum Beach, Sunshine Coast, Queensland (QLD), Australia

**Keywords:** Pandemics, Problematic Smartphone usage, Addictions, Neck pain, Social networking

## Abstract

**Background:**

Smartphone use has increased significantly, especially during the period of global pandemic caused by the novel SARS-CoV2 coronavirus (COVID-19). Concurrently, smartphone addiction is a growing social problem in children and adolescents with the consequence of adverse health outcomes. This study assessed the prevalence of smartphone addiction, patterns of use, and the experienced body-region discomfort among Iranian school students during the COVID-19 pandemic.

**Methods:**

A cross-sectional study with students from grades 1–9 recruited *n* = 585 participants (mean age = 14.49 (2.26 years); female = 65.8%). Data were collected from parents and students through the online 'Smartphone addiction scale-short version’ (SAS-SV), self-reported demographic questionnaires, and extracts of the Nordic musculoskeletal questionnaire for the evaluation of musculoskeletal disorders.

**Results:**

The prevalence rate of smartphone addiction (53.3%) was relatively high in the overall sample. Participants spent 6.85 (4.62) hours per day on their smartphones, which had increased 53.86% relative to the pre-pandemic period. The primary smartphone uses were for social networking (77.9%), web-surfing (53.3%), and camera activities (50.9%). There was a positive correlation between smartphone addiction as assessed with the SAS-SV and daily use time (*r* = 0.34, *p* < 0.001), and the percentage of change relative to the pre-pandemic period (*r* = 0.26, *p* < 0.001). Discomfort related to smartphone use was mostly reported as present in the eyes (39.7%) and neck (39.1%). A positive correlation was found (*p* < 0.001) between smartphone addiction and discomfort in the eyes, neck, wrists, shoulders, and upper-back.

**Conclusion:**

The more frequent usage of smartphones by students during the Covid-19 pandemic were associated predominantly with discomfort to the eyes and neck. Parents should consider the complications of musculoskeletal and postural changes during the child’s future years and pay particular attention to the individual’s patterns of smartphone use with an emphasis on posture and usage that reduces discomfort to the eyes and the musculoskeletal system, particularly the neck.

## Introduction

Smartphone technology has become an integral part of life for its numerous benefits [1]. Smartphones users are throughout the globe, across all social and economic divides, for multiple different situations and purposes, and across all ages of adults, adolescents and children [2]. As with adult trends, childhood smartphone use has increased dramatically in many countries worldwide. Iran is the world’s thirteen highest smartphone user with over 52 million users in 2021 [3]. Despite the advantages, various negative smartphone consequences affect physical and mental health, reduce social interaction, cognitive disorders, vision, sleep quality, sedentary lifestyle, obesity and psychological dependence which includes smartphone addiction in children [4–6].

The novel SARS-CoV2 coronavirus (COVID-19) was initially reported in China, then spread to create a global pandemic that resulted in changes in the way we are living our lives [7]. Many governments imposed lockdown to break the chain of virus transmission which required individuals and families to stay at and work from home [8]. The unexpected disruption of social interactions led to physical behavioral and mental health changes for all individuals, including children [9]. Children were required to stay at home and spend more time using technological devices such as smartphones or tablets for school learning and entertainment [10–12]. This led to the restricted mobility and daily activities [13], less physical activity [14], interrupted sleep patterns [12], overuse of smartphones, and to potentially render a pathological effect with resulting smartphone addictions [15]. Apart from the psychological negative effects, excessive exposure to smartphones can also result in poor vision [4], musculoskeletal pain in the neck, as well as in the wrist and fingers. The physical consequences of smartphone overuse during the pandemic duration are often ignored [5].Prevalence rates of smartphone addiction in children and adolescents is reported from 5% [16] to 50% pre-pandemic [17]. Anecdotally, smartphone dependency has increased within the short timeframe of the pandemic when compared to pre-COVID. Smartphones are used as the main tool for connecting to the internet, and a child’s age at first internet use is the main risk for smartphone addiction and related mental and physical health problems [18]. Stressful periods of confinement during the COVID-19 pandemic were associated with smartphone overuse in relation to different aspects of the children’s lives. This included social interaction, web surfing, gaming, and instant messaging, all of which can potentially create psychological and physical disorders [13]. To our knowledge, there is has been no study that has directly monitored the rate and pattern of smartphone use, and its effects on the MSDs of children in Iranian.

Consequently, this study’s aim was to evaluate: the prevalence rate of smartphone addiction among Iranian children during the COVID-19 pandemic; compare the rate of use with the pre-pandemic period; evaluate the pattern of use; and measure the experienced discomforts.

## Methods

### Participants and procedure

This cross-sectional study recruited 585 school-age children (200 males and 385 females, age 6–19 years) was completed between April 2021 and January 2022. During this period Iran was within the fourth and fifth waves of the COVID-19 pandemic and the consequential lockdowns. Inclusion criteria were that participants have their own smartphone and a history of smartphone use that was > 1 year. Written informed consent was obtained from all children and their parents prior to the completion of the study’s questionnaires and survey. The study was approved by the Ethics Committee of the University of Social Welfare and Rehabilitatin Sciences (USWR), Iran, Tehran (IR.USWR.REC.1400.189).

**Fig. 1:**
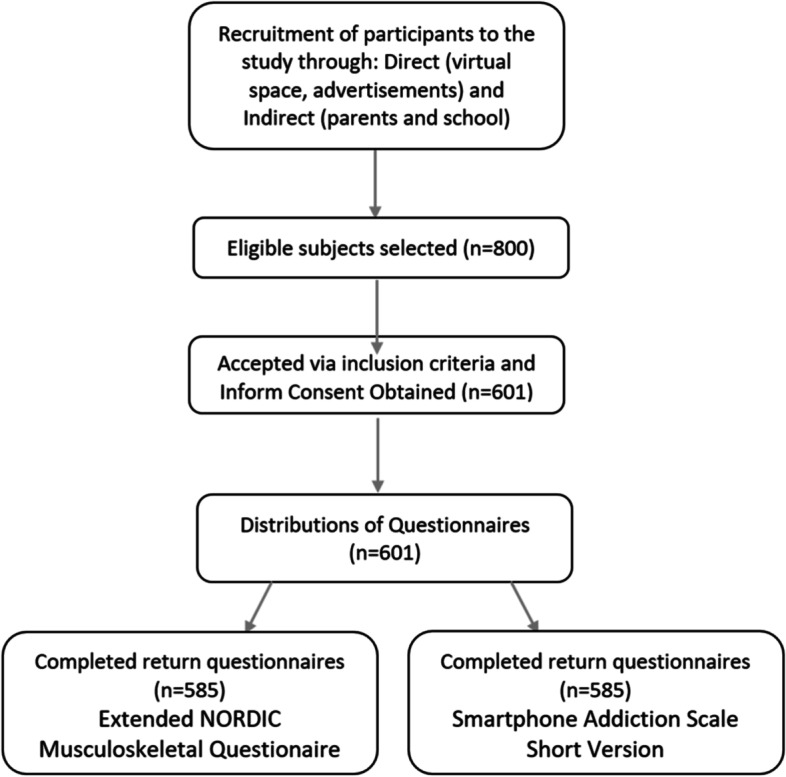
Flow chart of the study process

Two self-report questionires were administered to collect data in the form of electronic-based survey questionnaires: one involved demographic information that included some aportioned aspects of the ‘Extended NORDIC Musculoskeletal Questionaire’ (NMQ) [19], the second was the Persian version of the smartphone addiction scale short version (SAS-SV) [20]. Data was collected using an online survey process by sharing the survey link: *directly* through the social media platforms of ‘Telegram’, ‘Whatsapp’ and also via directl email to children in middle and upper highschool; and *indirectly*, through parents of primary school children (Fig. 1).

The raw data was exported in a Comma-separated values (CSV) format to be recorded in an Excel Workbook then entered into the SPSS statistical program for analysis.

The sample size required to ensure adequate power analysis, was calculated from the equation:

$$n=\frac{pq{\left({z}_{1-\frac{a}{2}}\right)}^{2}}{{\left(d\right)}^{2}}=\frac{0.2275{\left(1.96\right)}^{2}}{{\left(0.04\right)}^{2}}=547$$

where *p* =An estimation of the proportion of addicted smart phone users in the population; *q*=(1-p), *α*=type I error, *d*= [the precision] and *z*=the standardized values from the ‘normalized distribution table’.

We assumed the prevalence of smartphone addiction, *p* = 0.35 (35%), (α = 0.05), and a precision (d) = 0.04. The calculated sample size was found to be *n* = 547, and then adjusted for a dropout rate of 10% to give the final sample size estimation *n* = 601. Based on the sampling framework and with respect to the existing student distribution in primary, middle and high school, this study sought *n* = 187, *n* = 195 and *n* = 219 students respectively in the three school levels for participation in the study. We distributed 601 questioners between the subjects but a non-response rate of *n* = 16 (2.6%) provided a final *n* = 585 for data extraction and statistical analysis which exceeded the minimum *n* = 547 required for adequate sample power.

### Questionnaires:

#### Demographics questionaire:

The demographic, physical discomfort and pattern of use characteristics were included in a single questionire with the following variables: age, gender, duration of daily smartphone use, school year, region of residence, aim of smartphone use, rate of smartphone use relative to the pre-COVID-19 pandemic, and experienced discomfort during smartphone use.

#### Smartphone Addiction Scale-Short Version (SAS-SV):

The SAS-SV is a validated self-report questionnaire extracted from the original SAS and used to screen for smartphone addiction. The SAS-SV has 10-items on a six-point Likert-scale (anchored at 1 = ‘‘strongly disagree’’ and 6 = ‘‘strongly agree’’), with a total score-range of 10–60 where a higher score indicates greater smartphone dependency [21]. The original short version reported a single factor structure with an addiction cutoff of 31-points for men and 33-points for women to identify the person at the risk of pathological smartphone dependency [20, 21].

### Statistical analysis

All data analysis was performed using the statistical program for social sciences (SPSS), Released 2007, Version 16.0 for Windows (Chicago, SPSS Inc). Descriptive statistics respectively included the mean and standard deviation (SD) values for continuous variables represented as mean (SD); with frequency and percentage applied for categorical variables. The Kolmogorov–Smirnov (K-S) test was conducted to test the normality of distribution. Pearson's chi-squared test was used to evlaute the correlation between MSDs and smartphone addiction. The correlation between the duration of daily use and the smartphone addiction was evaluated by Pearson correlation coefficient. An independent t-test was conducted to confirm the differences in the main variables according to gender and age. A *p* value < 0.05 was considered statistically significant.

## Results

Demographics for the recruited children [*n* = 585, female = 65.8%, mean-age = 14.49 (2.26) years], 182 (31.1%) primary school students aged 6–11 years, 190 (32.5%) middle school students aged 12–14 years, and 213 (36.4%) high schoool students aged 15–19 years. All data was normally distributed (*p* > 0.11) enabling the use of parametric statistical tests.

Participants smartphone use during the COVID-19 pandemic was 6.85 (4.62) hours per day. They reported an average time per day of smartphone use increase during the pandemic of 53.86% relative to the pre-pandemic period (Table 1).

**Table 1:** General characteristics of the groups

Sex	Female *n* (%)	385 (65.8)
	Male *n* (%)	200 (34.2)
School level	Primary *n* (%)	182 (31.1)
	Middle *n* (%)	190 (32.5)
	High *n* (%)	213 (36.4)
Age (years) Mean (SD)	Overall	14.49 (2.26)
	Primary (6–11)	9.7 (1.16)
	Middle (12–14)	13.35 (0.75)
	High (15–19)	16.14 (1.12)
Smartphone use per day (hours)		6.85 (4.62)
Increase pecentage use after pandemic (%)		53.86
SAS-SV Score Mean(SD)	Overall	33.68 (12.40)
	Male	33.76 (12.63)
	Female	33.64 (12.29)
SAS score/Education Mean (SD)	Primary school	35.42 (12.32)
	Middle school	33.99 (13.14)
	High school	33.15 (11.88)

*SD* Standard Deviation, *SAS* Smartphone Addiction Scale, *SV* Short Version

The top three reasons reproted for smartphone use were: social networking (77.9%), web surfing (53.3%), and camera use (50.9%). The patten of gender-based use is presented in Table 2.

**Table 2:** Pattern of smartphone use in overall sample and gender base charecters

Pattern of use	Phone call	Web surfing	Email	Game	News	Social network	Camera	Others
Overall sample N (%)	263 (45)	312 (53.3)	44 (7.5)	234 (40)	96 (16.4)	456 (77.9)	298 (50.9)	201 (34.4)
Gender:								
Male	96 (48)	123 (61.5)	22 (11)	121 (60.5)	47 (23.5)	153 (76.5)	82 (41)	59 (29.5)
Female	167 (43.4)	189 (49)	22 (5)	113 (29.3)	49 (12.7)	303 (78.7)	216 (56.1)	142 (37)

The most prevalent regions of discomfort were the eye (39.7%) and neck (39.1%). The prevalence of smartphone addiction was 53.3% (*n* = 312) in the overall sample; 54.5% in males (*n* = 109), and 52.7% (*n *= 203) in females. The SAS-SV scores were not significantly different between males and females (*p* = 0.91). The prevalence of addiction in the categories of primary, middle and high school were respectively 63.2% (*n* = 115), 53.6% (*n* = 102) and 51.4% (*n* = 109).

There was a positive corelation analysis between smartphone addiction and daily use (*r* = 0.34, *p* < 0.001) and the percentage of change relative to the pre-COVID-19 period (*r* = 0.26, *p* < 0.001). The chi-square results showed a significant relation between smartphone addiction and experienced discomfort (Table 3) in the neck, upper-back, shoulders, eyes and wrist (*p *< 0.001).

**Table 3:** Correlation between smartphone addiction and experienced discomfort

Regions	Discomfort level	Addiction levels (n)		Pearson Chi square value	Significance	Contingency Coefficient
		Addicted	No addicted			
Wrist	Yes	62	28	10.34	0.001	0.133
	No	250	245			
Fingers	Yes	50	30	3.12	0.070	0.077
	No	262	243			
Shoulders	Yes	67	24	17.83	< 0.001	0.175
	No	245	249			
Neck	Yes	76	154	29.27	< 0.001	0.112
	No	158	198			
Upper-Back	Yes	80	28	22.89	< 0.001	0.243
	No	232	245			
Eyes	Yes	146	86	14.23	< 0.001	0.154
	No	166	187			

## Discussion

This study aimed to reveal the prevalence of smartphone addiction, the smartphone pattern of use, and to identify the regions of experienced discomfort in school children during the COVID-19 pandemic. This is the first study in Iran involving school students’ smartphone addiction during the COVID-19 pandemic.

Our results demonstrated that during the pandemic, children significantly increased their average daily smartphone use. The SAS-SV score showed a prevalence of addiction in the overall sample of 53.3% where the most affected students were from primary school (63.2%), then middle school (53.6%), and lastly high school (51.4%). These findings are higher than those reported in previous studies [22–25]. Based on our results, over half of the students suffered from smartphone addiction during the COVID-19 pandemic, which is significantly higher than the reported overall prevalence in the general population. The rate of smartphone addiction prevalence in adolescents has been reported in the Philippines (21%) [24], Hong Kong (18%) [24] and England (10%) [22]. Further, our results also showed a higher addiction prevalence than that found in medical students in India (24.65%) [26], Poland (37.02%) [27], and Spain (14.9%) [28].

The high prevalence of smartphone addiction in our study is related to the period of the COVID-19 pandemic, where as that collected in previous studies was gathered pre-pandemic [4, 29, 30].

The lockdown during the pandemic disrupted daily routines [31] and forced children to stay at home to perform school tasks and online courses using their smartphone. At the time of social separation, students relied on the useful aspects of the smartphone, such as social communication, information processing, and learning, to a greater degree than they had in normal pre-pandemic circumstances [32]. Further, due to the partial closure of schools, the simplest device for connecting to the internet was the smartphone, which was also dominat in their social life connections through the various available social media platforms [33–35]. New learning strategies were also possible through the medium of the smartphone which further increased use and conseqeuntly contributed to the determined higher degree of addiction. With the start of the pandemic, recreational and other source of entertainment were limited [31] and students therefore found their smartphone as one of the only sources of entertainment [34]. The high prevalence of smartphone addiction in Iranian school students can be interpreted as a direct relation to the the social and academic environment that prevailed during the pandemic COVID-19 period.

Our results showed that the subjects spent more than 6-h per day using their smartphone. It is recognised that the likelihood of smartphone addiction will be increased by the duration of daily use. Previous studies in Bangladesh and Hungary [36, 37] reported that a 2-h cut-off time interval be used as the predecessor of addiction. Further studies have shown that > 6 h of daily internet use is an independent risk factor for internet addiction [38–40]. Studies in Turkey and the Mediteranean region [41, 42] have also shown that there was already > 3 h daily internet use in children of the ages within our study before the pandemic began, and that this time-use increased during the pandemic. As with previous studies [26, 30, 40, 43], we also found a direct correlation between the time spent on the smartphone and the presence of addiction.

Previous research has suggested a correlation between internet addiction and the level of social media use [44], online gaming, and web-surfing [40, 45]. In our study, the most prevalent aim of smartphone use reported by the participating students was that of social networking (77.9%) then web-surfing (53.3%). This is consistent with the previious findings that reported the main reasons for smartphone use as calling parents and friends (96%), social networking (91%), and school-related study use (78%) [25]. A further study detailed the leading smartphone uses among Phillipines high-school students were accessing social network sites (50.4%), online chatting (16.7%), and gaming (11.3%) [46], again corroborating the findings of this study.

The prevalence of smartphone addiction in males was found to be higher than for females, but this was not significant. The possibe explanation may be the known lower cutoff-scores for males compared to females based on previous SAS-SV research [21]. Our results conseqeuntly did not confirm the hypothesis of a relation between gender and smartphone addiction. This finding has been shown in previous research [21, 47–49]. In contrast, our results are not consistent with studies that reported smartphone addiction to be significantly related to gender [23, 50, 51]. To explore these discrepancies in findings related to gender we can consider the pattern of gender use. Our study found the prevalent purposes of smartphone use: in females were social networking, web-surfing and camera use; while for males it was also social networking and web-surfing, but gaming was was also noted. Consequently, the pattern of general and total use, though relatively similar across genders, may be related to specific circumstances of the pandemic and the specific choice of use patterns and options. It appears that further studies should be conducted to cnsider the inconsistency in gender related results and the prevalence of smartphone addiction.

Addicted subjects showed greater MSD discomfort compared to non-addicted subjects. Smartphone overuse is reported in the literature as usually being associated with neck, shoulders and wrist dsicomfort [52]. This can be a consequence of factors such as improper smartphone screen display size, static postures, a high number of messages being sent, sedentary lifestyles, daily hours spent on the smartphone, decreased in physical activity (PA) [31] and the self-selected awake posture [52–54]. It has been shown that home confinement results in a marked reduction in PA and consequently cardiovascular fitness, and that both aspects affect the musculoskeletal system [31].

This is in agreement with the findings of our study, in which neck pain was the highest concern reported by participants. Another commonly reported physical discomfort is that of ocular problems, such as dry eye disease and fatigue [40, 52]. In the current study, 39.7% of participants reported dry eyes and fatigue after smartphone overuse, a finding similar to previously reported research [40, 52].

### Strengths and limitations

Some limitations in the current study are related to the cross-sectional nature of the research, which needs to be considered when interpreting and generalizing the findings. As this study was conducted during the COVID-19 pandemic, generalizability is limited in relation to general or non-lockdown periods. Further, the causal relationship between smartphone addiction and experienced discomfort cannot be definitively established. Additionally, the consequences of smartphone addiction are beyond the measured variables of this study and longitudinal or follow-up research would be required to determine the affect on biopsychosocial health status. As the age of the smartphone user is gradually decreasing, future studies should consider the evaluation of the consequences of smartphone overuse through multiple approaches that account for both the physical and psychosocial consequences of the individual users.

Some strenghts in the current study are related to this being the first such study in a Persian population, that it reflects differences in usage preferences by gender that do not alter the overall useage prevelance, and that the findings support work from various different regions in previous research, which together provide an updated undersetanding and progression of the knowledge within this field.

## Conclusions

This study demonstrated an increased smartphone addiction and prevalence among Iranian school children during the COVID-19 pandemic when compared to pre-epidemic findings. The students used the smartphone more than 6-h per day, which is a risk for their health status. The most common reasons for smartphone usage were reported as being for social communication, camera, web-surfing and gaming. However, though the primary aims of smartphones usgae during this pandemic period were learning strategies, performing school tasks, and maintaining social interaction, it appears that other patterns of use had become dominant. Concerns about the diverse health effects should be considered due to the obtained correlation between the duration of smartphone use and the experienced discomfort in different body regions such as the neck, wrist and eyes. Parents should be aware of the adverse effects of overuse and inappropriate use of smartphones by their children. Parents should consider how they control and monitor the pattern of smartphone usage in order to prevent the risks of musculoskeletal disorders being created. By incorporating other physical and recreational activites in the childrens daily program, a preventative effect can be provided that reduces the smartphone overuse and consequently its subseqeunt affects on the childrens’ health.

## Data Availability

The datasets used during this study are available from the corresponding author on reasonable request.

## Abbreviations

NMQ: Nordic musculoskeletal questionnaire
MSDs: Musculoskeletal disorders
SAS-SV-Pr: Smartphone Addiction Scale-Persian short Version
SD: Standard deviation
CSV: Comma-separated values format
